# Measuring Adoption of Patient Priorities-Aligned Care Using Natural Language Processing

**DOI:** 10.1093/geroni/igaa057.592

**Published:** 2020-12-16

**Authors:** Javad Razjouyan, Jennifer Freytag, Edward Odom, Lilian Dindo, Aanand Naik

**Affiliations:** 1 Center for Innovations in Quality, Effectiveness, and Safety (iQuest), Houston, Texas, United States; 2 Michael E. DeBakey VA Medical Center, houston, Texas, United States; 3 Michael E. DeBakey VA Medical Center, Houston, Texas, United States

## Abstract

Patient Priorities Care (PPC) is a model of care that aligns health care recommendations with priorities of older adults with multiple chronic conditions. Social workers (SW), after online training, document PPC in the patient’s electronic health record (EHR). Our goal is to identify free-text notes with PPC language using a natural language processing (NLP) model and to measure PPC adoption and effect on long term services and support (LTSS) use. Free-text notes from the EHR produced by trained SWs passed through a hybrid NLP model that utilized rule‐based and statistical machine learning. NLP accuracy was validated against chart review. Patients who received PPC were propensity matched with patients not receiving PPC (control) on age, gender, BMI, Charlson comorbidity index, facility and SW. The change in LTSS utilization 6-month intervals were compared by groups with univariate analysis. Chart review indicated that 491 notes out of 689 had PPC language and the NLP model reached to precision of 0.85, a recall of 0.90, an F1 of 0.87, and an accuracy of 0.91. Within group analysis shows that intervention group used LTSS 1.8 times more in the 6 months after the encounter compared to 6 months prior. Between group analysis shows that intervention group has significant higher number of LTSS utilization (p=0.012). An automated NLP model can be used to reliably measure the adaptation of PPC by SW. PPC seems to encourage use of LTSS that may delay time to long term care placement.

